# Supplementary material for: Innate extracellular vesicles from melanoma patients suppress β-catenin in tumor cells by miRNA-34a
Source: Life Sci Alliance. 2019 Mar 7;2(2):e201800205. doi: 10.26508/lsa.201800205 (PMC6406044; doi:10.26508/lsa.201800205)
Supplement: Supplementary file 1 [file LSA-2018-00205_TableS1.docx]

**Table S1**

| **Fig. 1A** | | **T1 – T14** | | | | | | | |
| --- | --- | --- | --- | --- | --- | --- | --- | --- | --- |
| **Fig. 1B** | | **T15** | | | | | | | |
| **Fig. 1C** | | **T1; T16; T17** | | | | | | | |
| **Fig. 1D** | | **T1; T11; T12; T18 - T26** | | | | | | | |
| **Fig. 1E** | | **T1; T11; T12; T16; T17; T18; T19; T20; T21; T22; T23; T24; T25; T26** | | | | | | | |
| **Fig. 1F** | | **T1 - T14** | | | | | | | |
| **Fig. 2A** | | **T1 - T14; HR1 - HR15; LR1 - LR11** | | | | | | | |
| **Fig. 2B** | | **T1 - T14; HR1 - HR15; LR1 - LR11** | | | | | | | |
| **Fig. 2C** | | **T1 - T14; HR1 - HR15; LR1 - LR11** | | | | | | | |
| **Fig. 2D** | | **HR16 - HR25; LR7; LR12 - LR21** | | | | | | | |
| **Fig. 2E** | | **T11; T12; T20 - 27; HR16 - HR25** | | | | | | | |
| **Fig. 2F** | | **T1; T11; T12; T18 - T26; HR16 - HR25; LR7; LR12 - LR21** | | | | | | | |
| **Fig. 2G** | | **T18 - T20; HR16 - HR18** | | | | | | | |
| **Fig. 3A** | | **T18 - T19; HR16 - HR18; LR20 - LR22** | | | | | | | |
| **Fig. 3B** | | **LTS1 – LTS2** | | | | | | | |
| **Fig. 4A** | | **T1 - T14** | | | | | | | |
| **Fig. 4C** | | **T1 - T14; HR1 - HR15; LR1 - LR11** | | | | | | | |
| **Fig. 5A** | | **T21 - T23; HR22 - HR24; LR17 - LR19** | | | | | | | |
| **Fig 5B** | | **T21 - T23; HR22 - HR24; LR17 - LR19** | | | | | | | |
| **Fig. 5C** | | **T24 - T26; HR22 - HR24; LR20 - LR22** | | | | | | | |
| **Fig. 5D** | | **T1 - T14; HR1 - HR15; LR1 - LR11** | | | | | | | |
| **Fig. 5E** | | **T1 - T14; HR1 - HR15; LR1 - LR11** | | | | | | | |
| **Fig. 6A** | | **T24 - T26; HR16 - HR18; LR12; LR13; LR23** | | | | | | | |
| **Fig. 6B** | | **T26; HR25; LR24** | | | | | | | |
| **Fig. 6C** | | **T16; T17; HR19 - HR21; LR14 - LR16** | | | | | | | |
| **Fig. 6D** | | **T16; T17; HR19 - HR21; LR14 - LR16** | | | | | | | |
| **Fig. 6E** | | **T21 - T23; HR22 - HR24; LR17 - LR19** | | | | | | | |
| **Fig. 7A** | | **T1 - T14; HR1 - HR15; LR1 - LR11** | | | | | | | |
| **Fig. 7B** | | **T1 - T14; HR1 - HR15; LR1 - LR11** | | | | | | | |
| **Fig. 7C** | | **T24-T26; HR19 - HR21** | | | | | | | |
| **Fig. 7D** | | **T24 - T26; HR22 - HR24** | | | | | | | |
| **Fig. S2A** | | **T27** | | | | | | | |
| **Fig. S2C** | | **T1 – T14; HR1 - HR15; LR1 - LR11** | | | | | | | |
| **Fig. S4A** | | **T25; HR6; LR6** | | | | | | | |
| **Fig. S4B** | | **T1 – T14** | | | | | | | |
| **Fig. S5** | | **HR16 - HR25** | | | | | | | |
| **Fig. S7** | | **T16; T19; T26; HR20; HR21; HR25; LR12; LR13; LR24** | | | | | | | |
|  | | | | | | | | | |
| **ID** | **gender** | **age** | **collection date** | **tumor thickness** | **clinical stage** | **primary tumor** | **metastases** | **R0 resection date** | **Figure ID** |
| T1 | w | 54 | 09.2011 | not measured | IV | no | yes | 03.1999 | 1A;1C; 1D;1E 1F; 2A; 2B; 2C; 4A; 4C; 5D; 5E; 7A; 7B; S2C; S4B |
| T2 | w | 73 | 12.2011 | 6.0 mm | IV | no | yes | 06.2010 | 1A; 1F; 2A; 2B; 2C; 4A; 4C; 5D; 5E; 7A; 7B; S2C; S4B |
| T3 | w | 61 | 03.2012 | not measured | III | no | yes | 02.2007 | 1A; 1F; 2A; 2B; 2C; 4A; 4C; 5D; 5E; 7A; 7B; S2C; S4B |
| T4 | m | 43 | 12.2011 | 5.1 mm | IIIB | no | yes | 04.2009 | 1A; 1F; 2A; 2B; 2C; 4A; 4C; 5D; 5E; 7A; 7B; S2C; S4B |
| T5 | w | 73 | 05.2011 | not measured | IV | no | yes | 04.1999 | 1A; 1F; 2A; 2B; 2C; 4A; 4C; 5D; 5E; 7A; 7B; S2C; S4B |
| T6 | m | 63 | 05.2012 | 3.2 mm | IIC | no | yes | 12.2011 | 1A; 1F; 2A; 2B; 2C; 4A; 4C; 5D; 5E; 7A; 7B; S2C; S4B |
| T7 | w | 72 | 05.2012 | 4.5 mm | IV | no | yes | 03.2001 | 1A; 1F; 2A; 2B; 2C; 4A; 4C; 5D; 5E; 7A; 7B; S2C; S4B |
| T8 | m | 65 | 05.2012 | 2.8 mm | IIB | yes | no | Primary | 1A; 1F; 2A; 2B; 2C; 4A; 4C; 5D; 5E; 7A; S2C; S4B |
| T9 | m | 69 | 02.2013 | not measured | IV | no | yes | 06.1992 | 1A; 1F; 2A; 2B; 2C; 4A; 4C; 5D; 5E; 7A; 7B; S2C; S4B |
| T10 | w | 79 | 02.2013 | 3.0 mm | IIB | no | yes | 02.2009 | 1A; 1F; 2A; 2B; 2C; 4A; 4C; 5D; 5E; 7A; 7B; S2C; S4B |
| T11 | m | 76 | 12.2013 | 4.5 mm | IV | no | yes | 03.2006 | 1A; 1D; 1E; 1F; 2A; 2B; 2C; 2E; 4A; 4C; 5D; 5E; 7A; 7B; S2C; S4B |
| T12 | m | 75 | 10.2013 | 0.4 mm | IV | no | yes | 09.2003 | 1A; 1D; 1E; 1F; 2A; 2B; 2C; 2E; 4A; 4C; 5D; 5E; 7A; 7D; S2C; S4B |
| T13 | m | 75 | 09.2013 | 2.4 mm | IV | no | yes | 09.2011 | 1A; 1F; 2A; 2B; 2C; 4A; 4C; 5D; 5E; 7A; 7B; S2C; S4B |
| T14 | m | 51 | 03.2014 | 2.8 mm | IIB | yes | no | 03.2014 | 1A; 1F; 2A; 2B; 2C; 4A; 4C; 5D; 5E; 7A; 7B; S2C; S4B |
| T15 | m | 28 | 03. / 06./ 07.2012 | 0.3 mm | IA | yes | no | 05.2012 | 1B |
| T16 | m | 57 | 07.2013 | Uvea | IV | no | yes | 02.1011 | 1C, 1E; 6C; 6D; S7 |
| T17 | m | 72 | 05.2014 | 0.9 mm | IIIB | no | yes | 01.2011 | 1C, 1E; 6C; 6D |
| T18 | w | 61 | 10.2013 | 10.5 mm | IIC | no | circ. Tumor cells | 08.2008 | 1D; 1E; 2G; 3A |
| T19 | m | 57 | 09.2013 | 1.3 mm | IV | no | yes | 07.2007 | 1D; 1E; 2E; 2G; 3A; S7 |
| T20 | m | 45 | 08.2013 | 5.0 mm | IV | no | yes | 02.2010 | 1D; 1E; 2E; 2G; 3A |
| T21 | m | 77 | 10.2013 | 3.0 mm | IV | no | yes | 08.2010 | 1D; 1E; 2E; 6E; 5A; 5B |
| T22 | w | 84 | 10.2013 | 0.85 mm | IV | no | yes | 05.2006 | 1D; 1E; 2E; 6E; 5A; 5B |
| T23 | w | 39 | 10.2013 | 1.7 mm | IV | no | yes | 06.2003 | 1D; 1E; 2E; 6E; 5A; 5B |
| T24 | m | 70 | 10.2013 | 1.45 mm | IV | no | yes | 08.2007 | 1D; 1E; 2E; 5C; 6A; 7C; 7D |
| T25 | m | 65 | 12.2013 | 0.9 mm | IB | yes | no | 12.2013 | 1D; 1E; 2E; 5C; 6A; 7C; 7D; S4A |
| T26 | m | 75 | 12.2013 | 4.3 mm | IV | no | yes | 10.2005 | 1D; 1E; 2E; 5C; 6A; 6B; 7C; 7D; S7 |
| T27 | m | 26 | 11.2013 (2x) | 0.2 mm | IA | yes | no | 11.2013 | S2A |
|  |  |  |  |  |  |  |  |  |  |
| HR1 | w | 65 | 06.2012 | 2.9 mm | IIA |  |  | 08.2010 | 2A; 2B; 2C; 4C; 5D; 5E; 7A; 7B; S2C |
| HR2 | m | 55 | 10.2012 | 0.65 mm | IIIB |  |  | 07.2003 | 2A; 2B; 2C; 4C; 5D; 5E; 7A; 7B; S2C |
| HR3 | m | 49 | 10.2012 | 2.6 mm | IIB |  |  | 06.2009 | 2A; 2B; 2C; 4C; 5D; 5E; 7A; 7B; S2C |
| HR4 | m | 86 | 09.2012 | 5.0 mm | IIC |  |  | 09.2010 | 2A; 2B; 2C; 4C; 5D; 5E; 7A; 7B; S2C |
| HR5 | m | 54 | 09.2012 | 3.5 mm | IIIB |  |  | 12.2008 | 2A; 2B; 2C; 4A; 4C; 5D; 5E; 7A; S2C |
| HR6 | m | 56 | 08.2012 | 2.7 mm | IIIA |  |  | 01.2008 | 1A; 1F; 2A; 2B; 2C; 4C; 5D; 5E; 7A; 7B; S2C; S4A |
| HR7 | m | 50 | 06.2012 | 0.5 mm | IV |  |  | 09.1993 | 2A; 2B; 2C; 4C; 5D; 5E; 7A; 7B; S2C |
| HR8 | w | 71 | 07.2012 | 3.7 mm | IIIC |  |  | 2001 | 2A; 2B; 2C; 4C; 5D; 5E; 7A; 7B; S2C |
| HR9 | w | 39 | 01.2013 | 2.7 mm | IIB |  |  | 07.2008 | 2A; 2B; 2C; 4C; 5D; 5E; 7A; 7B; S2C |
| HR10 | m | 77 | 10.2012 | 1.9 mm | IIIA |  |  | 04.2006 | 2A; 2B; 2C; 4C; 5D; 5E; 7A; 7B; S2C |
| HR11 | m | 16 | 07.2012 | 2.4 mm | IIA |  |  | 06.2012 | 2A; 2B; 2C; 4C; 5D; 5E; 7A; 7B; S2C |
| HR12 | w | 48 | 07.2012 | 7.0 mm | IIIB |  |  | 06.2012 | 2A; 2B; 2C; 4C; 5D; 5E; 7A; 7B; S2C |
| HR13 | w | 67 | 09.2012 | 8.0 mm | IIC |  |  | 08.2012 | 2A; 2B; 2C; 4C; 5D; 5E; 7A; 7B; S2C |
| HR14 | m | 65 | 02.2014 | 6.0 mm | IV |  |  | 02.2006 | 2A; 2B; 2C; 4C; 5D; 5E; 7A; 7B; S2C |
| HR15 | w | 50 | 05.2012 | unknown primary | IIIC |  |  | 2000 | 2A; 2B; 2C; 4C; 5D; 5E; 7A; 7B; S2C |
| HR16 | w | 73 | 02.2013 | 3.1 mm | IIA |  |  | 05.2012 | 2D, 2E; 2G; 3A; 6A; S5 |
| HR17 | w | 72 | 03.2013 | 3.2 mm | IIB |  |  | 06.2012 | 2D, 2E; 2G; 3A; 6A; S5 |
| HR18 | m | 59 | 04.2013 | 3.2 mm | IIA |  |  | 09.2012 | 2D, 2E; 2G; 3A; 6A; S5 |
| HR19 | w | 66 | 05.2013 | 4.0 mm | IIC |  |  | 11.2006 | 2D, 2E; 6C; 6D; 7C; S5 |
| HR20 | m | 55 | 07.2013 | 1.2 mm | IV |  |  | 11.2007 | 2D, 2E; 6C; 6D; 7C; S5; S7 |
| HR21 | w | 72 | 05.2013 | 5.2 mm | IIIB |  |  | 11.2005 | 2D, 2E; 6C; 6D; 7C; S5; S7 |
| HR22 | m | 74 | 01.2013 | 8.0 mm | IIC |  |  | 08.2011 | 2D, 2E; 5A; 5B; 5C; 6E; 7D; S5 |
| HR23 | w | 23 | 02.2013 | 4.2 mm | IIB |  |  | 02.2007 | 2D, 2E; 5A; 5B; 5C; 6E; 7D; S5 |
| HR24 | w | 67 | 08.2012 | 3.0 mm | IIA |  |  | 06.2009 | 2D, 2E; 5A; 5B; 5C; 6E; 7D; S5 |
| HR25 | w | 36 | 01.2013 | 1.7 mm | IIIB |  |  | 08.2005 | 2D, 2E; 6B; S5 |
|  |  |  |  |  |  |  |  |  |  |
| LR1 | m | 77 | 03.2012 | 1.2 mm | IB |  |  | 07.2010 | 2A; 2B; 2C; 4C; 5D; 5E; 7A; 7B; S2C |
| LR2 | m | 51 | 01.2013 | 1.3 mm | IA |  |  | 05.2006 | 2A; 2B; 2C; 4C; 5D; 5E; 7A; 7B; S2C |
| LR3 | w | 74 | 01.2013 | 0.5 mm | IA |  |  | 11.2007 | 2A; 2B; 2C; 4C; 5D; 5E; 7A; 7B; S2C |
| LR4 | m | 45 | 11.2012 | 1.4 mm | IB |  |  | 10.2004 | 2A; 2B; 2C; 4C; 5D; 5E; 7A; 7B; S2C |
| LR5 | m | 72 | 11.2012 | 0.7 mm | IA |  |  | 02.2007 | 2A; 2B; 2C; 4C; 5D; 5E; 7A; 7B; S2C |
| LR6 | w | 43 | 02.2013 | 0.65 mm | IA |  |  | 05.1996 | 2A; 2B; 2C; 4C; 5D; 5E; 7A; 7B; S2C; S4A |
| LR7 | w | 47 | 02.2013 | 0.6 mm | IA |  |  | 07.2010 | 2A; 2B; 2C; 2D; 4C; 5D; 5E; 7A; 7B; S2C |
| LR8 | m | 52 | 02.2013 | 0.45 mm | IA |  |  | 07.2012 | 2A; 2B; 2C; 4C; 5D; 5E; 7A; 7B; S2C |
| LR9 | w | 57 | 02.2013 | 0.5 mm | IA |  |  | 07.2009 | 2A; 2B; 2C; 4C; 5D; 5E; 7A; 7B; S2C |
| LR10 | m | 52 | 10.2012 | 0.65 mm + 0.2 mm | IA |  |  | 05.2012 | 2A; 2B; 2C; 4C; 5D; 5E; 7A; 7B; S2C |
| LR11 | m | 37 | 10.2012 | 0.35 mm | IA |  |  | 03.2011 | 2A; 2B; 2C; 4C; 5D; 5E; 7A; 7B; S2C |
| LR12 | w | 72 | 10.2014 | 0.9 mm | IA |  |  | 08.2011 | 2D; 6A; S7 |
| LR13 | m | 51 | 10.2014 | 0.6 mm | IA |  |  | 09.2008 | 2D; 6A; S7 |
| LR14 | w | 44 | 10.2014 | 1.25 mm | IB |  |  | 05.2013 | 2D; 6C; 6D |
| LR15 | w | 61 | 10.2014 | 0.7 mm | IB |  |  | 11.2013 | 2D; 6C; 6D |
| LR16 | m | 45 | 10.2014 | 0.5 mm | IB |  |  | 10.2010 | 2D; 6C; 6D |
| LR17 | w | 31 | 05.2013 | 1.0 mm | IA |  |  | 03.2003 | 2D; 5A; 5B; 6E |
| LR18 | m | 36 | 04.2013 | 0.35 mm | IA |  |  | 11.2010 | 2D; 5A; 5B; 6E |
| LR19 | w | 57 | 03.2013 | 0.35 mm | IA |  |  | 02.2006 | 2D; 5A; 5B; 6E |
| LR20 | m | 51 | 10.2014 | 0.6 mm | IA |  |  | 08.2008 | 2D; 3A; 5C |
| LR21 | m | 49 | 02.2014 | 0.2 mm | IA |  |  | 01.2013 | 2D; 3A; 5C |
| LR22 | w | 35 | 01.2013 | 0.6 mm | IA |  |  | 06.2002 | 2D; 3A; 5C |
| LR23 | w | 24 | 05.2013 | 1,4mm | IB |  |  | 12.2012 | 2D; 3A; 5C; 6A |
| LR24 | w | 44 | 09.2015 | 0.9mm | IA |  |  | 08.2015 | 6B; S7 |
|  |  |  |  |  |  |  |  |  |  |
| LTS1 | w | 46 | 05.2014 | 0.9 mm | IV |  |  | 09.1999 | 3B |
| LTS2 | w | 62 | 06.2014 | 5.5 mm | IV |  |  | 05.1999 | 3B |
