# Supplementary material for: Innate extracellular vesicles from melanoma patients suppress β-catenin in tumor cells by miRNA-34a
Source: Life Sci Alliance. 2019 Mar 7;2(2):e201800205. doi: 10.26508/lsa.201800205 (PMC6406044; doi:10.26508/lsa.201800205)
Supplement: Supplementary file 2 [file LSA-2018-00205_TableS2.docx]

**Table S2**:

| **miRNA** | **F** | **p** | **adjusted p** |
| --- | --- | --- | --- |
| hsa-miR-3907 | 11,83 | 5,24E-06 | 2,87E-03 |
| hsa-let-7a-5p | 8,12 | 1,60E-04 | 2,87E-02 |
| hsa-miR-483-5p | 7,72 | 2,36E-04 | 2,87E-02 |
| hsa-let-7e-5p | 7,63 | 2,60E-04 | 2,87E-02 |
| hsa-miR-16-5p | 7,55 | 2,80E-04 | 2,87E-02 |
| hsa-miR-765 | 7,28 | 3,67E-04 | 2,87E-02 |
| hsa-let-7g-5p | 7,17 | 4,12E-04 | 2,87E-02 |
| hsa-miR-15b-3p | 7,15 | 4,20E-04 | 2,87E-02 |
| hsa-miR-342-3p | 6,96 | 5,11E-04 | 3,07E-02 |
| hsa-miR-150-5p | 6,81 | 5,94E-04 | 3,07E-02 |
| hsa-let-7b-5p | 6,74 | 6,43E-04 | 3,07E-02 |
| hsa-miR-92a-3p | 6,63 | 7,17E-04 | 3,07E-02 |
| hsa-miR-1181 | 6,53 | 7,92E-04 | 3,07E-02 |
| hsa-let-7i-5p | 6,48 | 8,36E-04 | 3,07E-02 |
| hsa-miR-199b-5p | 6,47 | 8,41E-04 | 3,07E-02 |
| hsa-miR-125a-5p | 6,41 | 8,99E-04 | 3,07E-02 |
| hsa-miR-550a-3p | 5,90 | 1,54E-03 | 4,70E-02 |
| hsa-miR-548q | 5,90 | 1,55E-03 | 4,70E-02 |
